# *FoMC69* Gene in *Fusarium oxysporum* f. sp. *radicis-lycopersici* Is Essential for Pathogenicity by Involving Normal Function of Chlamydospores

**DOI:** 10.3390/pathogens11121433

**Published:** 2022-11-28

**Authors:** Kazunori Sasaki, Yumi Ito, Yuki Hamada, Ayano Dowaki, Sudisha Jogaiah, Shin-ichi Ito

**Affiliations:** 1 Department of Biological and Environmental Sciences, Graduate School of Sciences and Technology for Innovation, Yamaguchi University, Yamaguchi 753-8515, Japan; 2 Research Center for Thermotolerant Microbial Resources (RCTMR), Yamaguchi University, Yamaguchi 753-8515, Japan; 3 Laboratory of Plant Healthcare and Diagnostics, PG Department of Biotechnology and Microbiology, Karnataka University, Dharwad 580003, India; 4 Department of Environmental Science, Central University of Kerala, Tejaswini Hills, Kasaragod 671316, India

**Keywords:** *Fusarium oxysporum* f. sp. *radicis-lycopersici*, tomato, chlamydospores

## Abstract

*Fusarium oxysporum* f. sp. *radicis-lycopersici* (Forl) causes crown and root rot disease in tomato, effecting severe economic losses. However, research on the pathogenicity genes and infection strategy of Forl is limited compared to that on *F. oxysporum* f. sp. *lycopersici* (Fol). In this study, we characterized *FoMC69* gene in Forl as a homolog of *MC69* required for pathogenicity in rice blast pathogen—*Magnaporthe oryzae*. Gene expression analysis revealed that *FoMC69* expressionin Forl is higher than that in Fol*in planta*. *FoMC69*-knockout mutant of Forl had significantly reduced root rot symptoms compared to the wild-type strain, and full pathogenicity was restored by complementation. By contrast, *ΔFoMC69* mutant of Fol presented the same symptoms as the wild type, suggesting that *FoMC69* of Forl, but not of Fol, was essential for full virulence in tomato plants. Morphological differences between the Forl and *ΔFoMC69* in the roots were observed by fluorescent labeling using WGA-FITC. Chlamydospores of the *ΔFoMC69* mutant of Forlcontinuously increased during infection and were three times higher than that of the wild type at 21 days post-inoculation. These observations suggest that *FoMC69* of Forl is required for virulence to tomato plants by involving the normal development and germination of chlamydospores.

## 1. Introduction

*Fusarium oxysporum* species complex is a soil-borne plant pathogenic fungus that infects more than 120 plants [1]. Based on host specificity, these fungi are classified as ‘*formae speciales* (f. sp.)’ [2]. *F. oxysporum* produces three types of asexual infectious spores, namely microconidia, macroconidia, and chlamydospores. Chlamydospores are formed by modification (wall thickening) of hyphal or conidial cells and are dormant, long-lived structures that can survive for several years [3,4]. The induction of chlamydospore formation is associated with stress factors, such as the absence of host plants, depletion of nutrients, and environmental conditions [5]. Chlamydospores germinate in response to host root exudates [6]. 

Tomato (*Solanum lycopersicum* L.) is the third most important vegetable crop worldwide, followed by potato and sugar beet [7]. Among *F. oxysporum* species, there are two tomato-infecting *formaespeciales*: *F. oxysporum* f. sp. *radicis-lycopersici* (Forl) and *F. oxysporum*f. sp. *lycopersici* (Fol)—the causative agents of Fusarium crown and root rot disease and Fusarium wilt disease, respectively. These two *formaespeciales*are difficult to distinguish morphologically, but can be identified based on symptom differences. Fusarium crown and root rot symptoms caused by Forl are yellowing, wilting, and severe root rot, whereas Fusarium wilt symptoms caused by Fol present yellowing, wilting, and vascular discoloration without root rot. Symptoms caused by Forl are enhanced by low temperatures (10–20 °C), waterlogged soil, saline water irrigation, low soil pH, and ammoniacal nitrogen. However, Fol symptoms are exacerbated by warm temperatures (∼28 °C), low soil pH, and the usage of ammonium-based fertilizer [3].

Plants have a two-layered immune system that prevents pathogen infection. The first layer is pattern-triggered immunity (PTI), whichis induced by the recognition of pathogen-associated molecular patterns (PAMPs), such as chitin by immune receptors [8]. However, plant pathogens secrete effector proteins into plant cells or apoplasts to facilitate invasion and proliferation, resulting in effector-triggered susceptibility (ETS) [8,9]. In the second layer, the plant directly or indirectly recognizes the effector by resistance (R) proteins, leading to effector-triggered immunity (ETI). ETI is a stronger immune response than PTI and induce a hypersensitive response or programmed cell death [10]. In the absence of effective R proteins, ETI is overcome, eventually leading to ETS [11]. With respect to Fol effectors, 14 Secreted in Xylem (SIX) proteins were identified in tomato xylem sap [12]. Moreover, of the *SIX* genes in *Fol*, *SIX1*, *SIX3, SIX5*, and *SIX6* are required for virulence in tomato plants [13,14,15,16]. However, in Forl*,* no *SIX* genes are included in the genome [13], and no genes responsible for pathogenicity, including effectors, have been documented.

While the effectors conserved only in specific species such as *SIX* genesare targeted susceptibility factors of specifichosts, conserved effectors across species called “core effector proteins” affectconserved defense mechanisms in plants [17]. MC69 is a small-secreted protein conserved in phytopathogenic fungi, including *F. oxysporum*, entomopathogenic fungi, fungal parasites, and saprophytic fungi [18]. These genes are related to the development of the invasion hyphae after appressorium formation in *Magnaporthe oryzae*, *Colletotrichum orbiculare*, and *C. graminicola* [18,19]. In this study, to identify the pathogenicity genes in Forl, we characterized *FoMC69* as a homolog of *MC69*, and the disrupted mutant showed a reduction in virulence. Further analysis showed that FoMC69 is related to the normal development or germination of chlamydospores during infection.

## 2. Materials and Methods

### 2.1. Fungal Isolates and Culture Conditions

The fungal strains used in this study were *F. oxysporum* f. sp. *radicis-lycopersici* MAFF 103007 and *F. oxysporum* f. sp. *lycopersici* CK3-1 [20]. The isolates were maintained on potato dextrose agar (PDA) at 4 °C. To produce conidia, the strains were cultured in a potato dextrose broth (PDB) at 25 °C for 7 days with shaking at 120 rpm.

### 2.2. Inoculation Test

Conidia were collected from the PDB culture and filtered through three layers of sterile gauze cloth to remove the mycelia. Spore concentrations were estimated using a hemocytometer and adjusted to 1 × 10^6^ conidia/mL. Tomato plants (‘Ponderosa’) were grown in pots containing vermiculite and perlite (1:1) mixture at 25 °C under a 12 h photoperiod with a photon flux density of 100 μmol∙m^−2^∙s^−1^ for 28 days. The roots of the 28-day-old plants were immersed in conidial suspension for 1 h. The inoculated plants were transferred to pots containing vermiculite and grown under pre-inoculation conditions. The root rot index of Forl inoculation was assessed by determining the root rot using the following scale: 0, no root rot symptom; 1, slightly damaged lateral roots; 2, browning of lateral roots but not main roots; 3, browning of both lateral and main roots; and 4, lateral and main roots are brown with lateral root collapse. The disease index of Fol inoculation was evaluated according to a previously described method [16]. For re-isolation of the fungi, root and stem sections obtained from the plant at 21 days post inoculation (dpi) were placed on Komada’s medium for *Fusarium* spp. [21] and cultured at 25 °C for 7 days. The experiments were repeated three times.

### 2.3. RNA Extraction and Quantitative Reverse Transcription Polymerase Chain Reaction

Roots and hypocotyls of the inoculated plants were harvested, frozen in liquid nitrogen, and stored at −80 °C until further use. Total RNA was extracted using Sepasol-RNA I Super G (NacalaiTesque, Kyoto, Japan). We performed cDNA synthesis using 1 µg total RNA in a 20-µL reaction volume with the ReverTraAce qPCR RT Master Mix with gDNA Remover (Toyobo, Osaka, Japan) according to the manufacturer’s instructions. The cDNA was diluted (1:1) and 1 µL was used as a template in a total volume of 20 µL of THUNDERBIRD SYBR qPCR Mix (Toyobo, Osaka, Japan). *FoMC69* gene-specific primers FoMC69-Q-F [5′-CCACAGTCTTTGTCACGCTTCT-3′] and FoMC69-Q-R [5′-AGCGCAACCTTGAGGAGTAACT-3′] were designed using Primer Express (Applied Biosystems, Foster City, CA, USA). *TEF* gene expression was used to normalize gene expression in each sample [22]. Quantitative PCR was performed using Quant Studio 1 (Applied Biosystems, Foster City, CA, USA). Relative quantification was performed using the ∆∆CT method [23].

### 2.4. Fungal Quantification

Fungal quantification was performed according to the method described by Fujikawa et al. [24] with minor modifications. For the quantification of Forl, specific primers CLP86-Q-F [5′-CTGCTGAGCCTGCTTCCAA-3′] and CLP86-Q-R [5′-TGTCACCTCCGCAGTCTTTAGA-3′] were designed using Primer 3 (http://bioinfo.ut.ee/primer3/, accessed on 6 October 2022) and used for quantitative PCR using THUNDERBIRD SYBR qPCR Mix.

### 2.5. Preparation of Gene Deletion and Complementation Constructs

A fusion PCR strategy was used to generate a gene knockout construct [25]. The 5′ and 3′ flanking regions of *FoMC69* were amplified using FoMC69-F1 [5′-ATGCCGGCGGATATGACGCTC-3′]/FoMC69-F2 [5′-GTCGTGACTGGGAAAACCCTGGCGGTTGATGATGCCGATGAT-3′] and FoMC69-F3 [5′-TCCTGTGTGAAATTGTTATCCGCTGTTGATTCGATTATAGTG-3′]/FoMC69-F4 [5′-CCAGGAAGATGCCCGAGTACG-3′] primers, respectively. The hygromycin B resistance gene (*hph*) cassette was amplified from the pHRC vector using the M13F/M13R primer set [26]. The three amplicons obtained were fused by fusion PCR using the FoMC69-F1/FoMC69-F4 primers. To generate a gene-complemented mutant, a DNA construct containing an open reading frame, upstream and downstream of *FoMC69*, was amplified using FoMC69-F1/FoMC69-F4 primer sets. The geneticin resistance gene cassette was amplified from the pII99 plasmid using M13F/M13R primer sets [27].

### 2.6. Fungal Transformation

For fungal transformation, polyethylene glycol (PEG)-mediated transformation was performed to generate gene knockout and gene complemented mutants. Protoplasts were prepared using methods described previously [28]. The protoplasts were resuspended in STC buffer and adjusted to 1.0 × 10^8^ protoplasts/mL. For the gene-knockout mutant, 20 µg of the construct was added to the protoplast suspension with 60% PEG solution [29]. For the gene complementation mutant, 10 µg of the complementation construct and 10 µg of the geneticin resistance gene cassette were co-transformed into fungal protoplasts. Transformants were selected and incubated on PDA plates containing hygromycin B (100 µg/mL) or G418 (100 µg/mL). Fungal DNA was extracted using a previously described simple extraction method [30]. Gene modification was verified by PCR using Quick Taq HS (Toyobo, Osaka, Japan) according to the manufacturer’s instructions.

### 2.7. Visualizing Fungal Colonization in Planta by WGA-FITC Labeling

Inoculated tomato roots were harvested and fixed in 50% ethanol for 1 h. The fixed tomato roots were immersed in 20% (*w*/*v*) KOH and incubated for 2 days. The roots were washed with PBS three times and stained with 0.2 µg/mL WGA-FITC (Lectin from *Triticum vulgaris*, Sigma-Aldrich, St. Louis, MO, USA) in PBS for 16 h in the dark. The stained root samples were observed using fluorescent microscopy BZ-9000 (Keyence, Osaka, Japan).

### 2.8. Quantification of Chlamydospores

The inoculated tomato roots were washed with water and fixed in FAA fixing solution for >24 h. The chlamydospores on the surface of the roots were counted using a microscope.

### 2.9. Statistical Analysis 

The experimental data are presented as the standard error of the mean. The statistical significance of differences between mean values was determined using Student’s *t*-test.

## 3. Results

### 3.1. Characterization of FoMC69 in Forl

**Figure 1:**
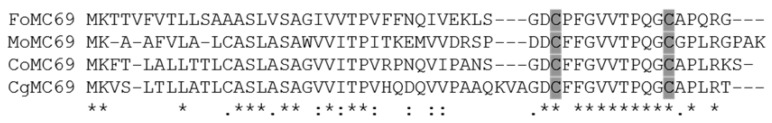
Amino acid sequence alignment of MC69 homologs. MC69 homologs sequence were obtained from the NCBI database. FoMC69: *Fusarium oxysporum* f. sp. *radicis-lycopersici* 26381 (EXL51339), MoMC69: *Magnaporthe oryzae* 70-15 (EHA46146), CoMC69: *Colletotrichum orbiculare* 104-T (AB669186), and CgMC69: *C. graminicola* M1.001 (XP_008093562). Two conserved cysteine residues are indicated in the gray box. Asterisks (*) indicate identical amino acids; Colons (:) indicate very similar amino acids, and dots (.) indicate similar amino acids.

To identify the *MC69* homolog in Forl, we performed a BLAST analysis with *MC69* of *M. oryzae* (*MoMC69*, EHA46146) as a query. We identified the *MC69* homolog (named *FoMC69*) in the Forl 26381 strain (accession No. EXL51339) with 44% similarity to *MoMC69*. *FoMC69* genes are expected to encode a protein of 54 amino acids (aa), and are 100% identical among the *F. oxysporum* species complex including Forl and Fol. SignalP 5.0 predicted a 19-aa signal peptide yielding a 35-aa mature protein. *FoMC69* has a conserved motif that contains two cysteine residues (Figure 1). *FoMC69* was identified as a single copy gene in the genome of the Forl MAFF 103007 and Fol CK3-1 strains by Southern blot analysis (Supplementary Figure S1).

**Figure 2:**
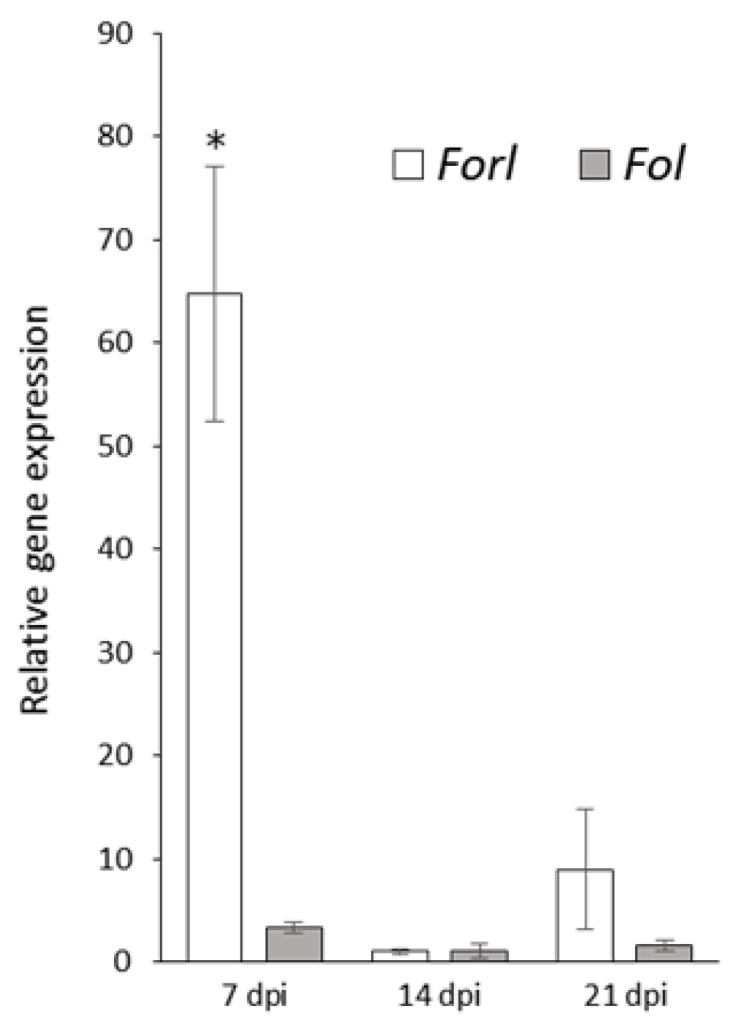
Expression of FoMC69 of Forl and Fol in tomato root tissues. Asterisk indicates significant differences (* *p* < 0.05) between Forl and Fol, as assessed by Student’s *t*-test.

Quantitative PCR analysis was performed to reveal the differential expression patterns of *FoMC69* between Forl and Fol. RNA was extracted from the roots inoculated with Forl or Fol at 7, 14, and 21 dpi. Little expression of *FoMC69* in Fol was confirmed (Figure 2). Incontrast, *FoMC69* expression in Forl was significantly higher than that in Fol (19.5-fold) at 7 dpi. At 14 and 21 dpi, the *FoMC69* expression level in Forl was reduced to that in Fol. 

### 3.2. FoMC69 Involved in Virulence of Forl but Not of Forl

**Figure 3:**
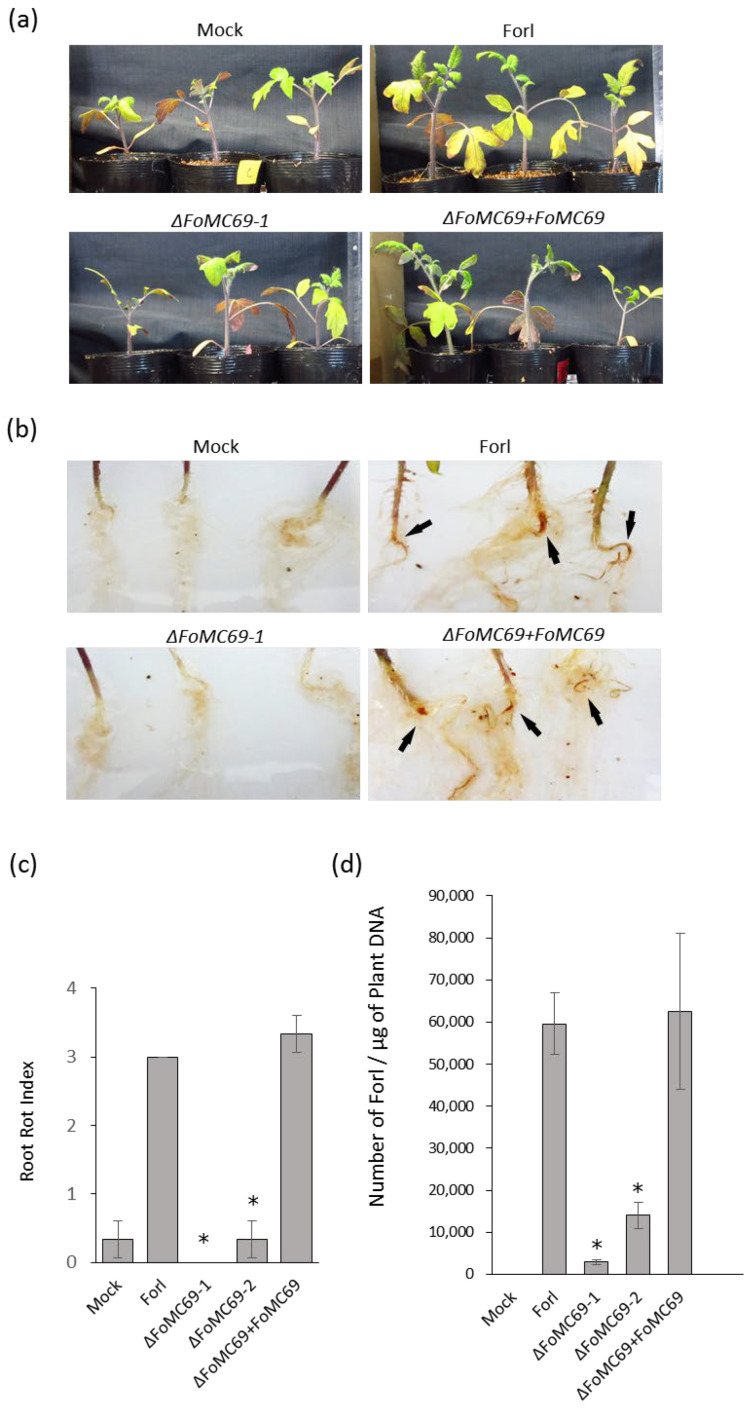
Inoculation test with *ΔFoMC69* of Forl against tomato plants at 21 days post inoculation. (**a**) Above-ground parts of tomato plants inoculated with Forl, *ΔFoMC69* mutant, and complementation strain. (**b**) Root rot symptoms when inoculated with Forl, *ΔFoMC69* mutant, and complementation strain. Arrows indicate root rot symptoms of the main roots. (**c**) Root rot index of tomato plants inoculated with Forl, *ΔFoMC69* mutant, and complementation strain. Asterisks indicate significant differences (*p* < 0.05) in the root rot index compared to that with wild-type Forl. (**d**) Number of Forl in the root of tomato plant inoculated with Forl, *ΔFoMC69* mutants, and complementation strain. Asterisks indicate significant differences (*p* < 0.05) in the number of cells compared to wild-type Forl, as determined by Student’s *t*-test.

To investigate whether *FoMC69* of Forl is involved in pathogenicity, this gene was knocked out from the Forl genome using a replacement strategy with a hygromycin resistance cassette as a selection marker (Supplementary Figure S2). The deletion of *FoMC69* was not affected fungal phenotype, radial growth, or conidial formation (Supplementary Figure S3). When *ΔFoMC69* mutants were inoculated into tomato plants, there were no symptoms in the leaf and stem parts as well as in the wild-type plants at 21 dpi (Figure 3a). The root parts inoculated with the wild type showed root rot symptoms in the main root, whereas *ΔFoMC69*-inoculated plants had no symptoms in the root, nor did mock plants (Figure 3b,c). The *ΔFoMC69-1* strain was complemented by the co-transformation of the *FoMC69* fragment and the geneticin resistance cassette. The complementation strain (*ΔFoMC69* + *FoMC69*) restored virulence, and presented root rot similar to that of the wild-type strain (Figure 3b,c). 

Colonization of the *ΔFoMC69* mutant in tomato tissues was investigated by incubating the root and hypocotyl samples on Komada’s medium. As a result, all strains (wild-type, deletion, and complemented strains) were recovered from root sections, but only the *ΔFoMC69* mutant was not detected in the hypocotyl. Therefore, the levelof fungal colonization in the roots was calculated using qPCR. The results showed that the number of *ΔFoMC69* strains in the roots was significantly lower than that in the wild-type and complementation strains at 21 dpi (Figure 3d).

**Figure 4:**
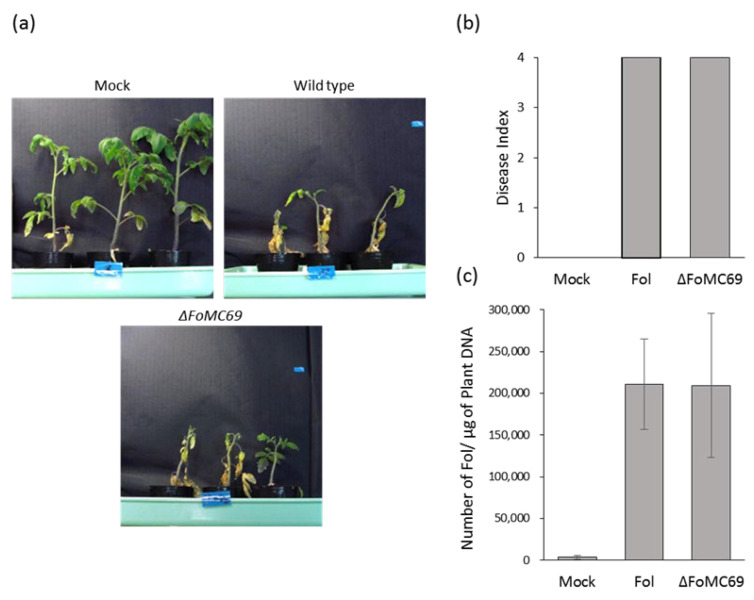
Inoculation test with *ΔFoMC69* of Fol against tomato plants. (**a**) Wilt symptom when inoculated with Fol and *ΔFoMC69* mutant. (**b**) Disease index of tomato plants inoculated with Fol and *ΔFoMC69* mutant. (**c**) Number of Fol in the tomato plant root inoculated with Fol and *ΔFoMC69* mutant.

*FoMC69* is also conserved in the genome of Fol (Supplementary Figure S1), and littleexpression of *FoMC69* in Fol compared with that in Forl was confirmed *in planta* (Figure 2). To investigate the pathogenicity of a *FoMC69* of Fol, *FoMC69*-disrupted mutant of Fol was generated and inoculated into tomato plants. The *ΔFoMC69* mutant of Fol caused severe disease symptoms similar to the wild-type strain at 21 dpi (Figure 4a,b). The fungal copy number in *ΔFoMC69*-inoculated root was almost identical to that in wild-type-inoculated roots (Figure 4c). 

### 3.3. Morphological Observation and Quantification of Chlamydospore in Planta

**Figure 5:**
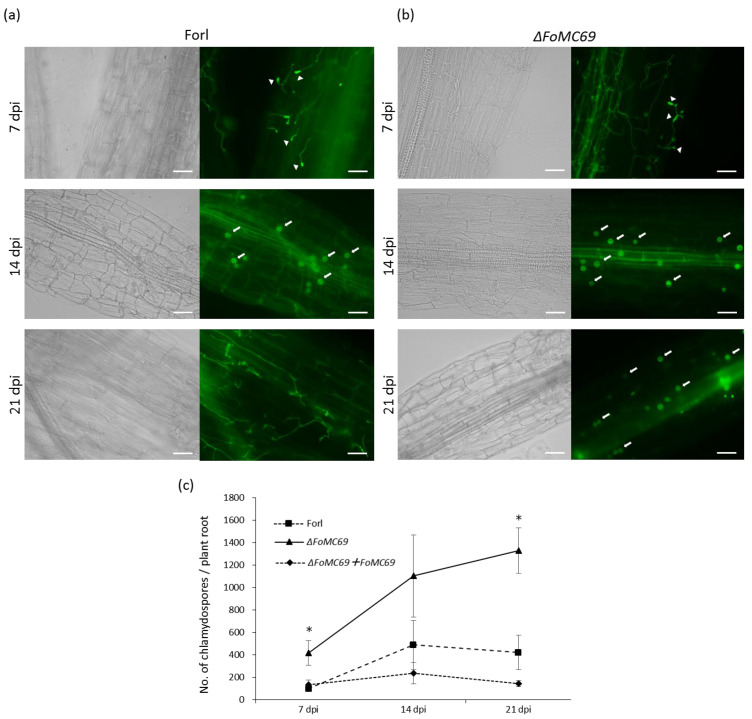
Observation and quantification of chlamydospore formation in the inoculated tomato root. (**a**,**b**) Morphology of Forl (**a**) and *ΔFoMC69* (**b**) labeling with WGA-FITC (green fluorescence) in tomato root at 7 dpi (top panels), 14 dpi (middle panels), and 21 dpi (bottom panels). The left panel shows bright field microscopy, while the right panel shows fluorescence microscopy. Arrowheads indicate geminated macroconidia. Arrows indicate chlamydospores. The bar is 20 µm. (**c**) Quantification of chlamydospores of Forl, *ΔFoMC69* mutants, and complementation strain in tomato roots. Asterisks indicate significant differences (*p* < 0.05) in the number of chlamydospores compared to that in the case of wild-type Forl, as assessed by Student’s *t*-test.

It has been reported that *ΔMC69* mutants of *M. oryzae* and *C. graminicola* did not promote the development of invasive hyphae after appressorium formation [18,19]. Therefore, the morphology of the *ΔFoMC69* mutant of Forl in the roots was observed using fluorescent labeling with WGA-FITC, which binds chitin. At 7 dpi, the germinated spores and elongating hyphae were observed in both Forl and *ΔFoMC69* strains (Figure 5a,b, top panel). Moreover, many chlamydospores were formed on the surface of the roots in both strains at 14 dpi (Figure 5a,b, middle panel). In the roots inoculated with the wild-type strain, chlamydospores were reduced and hyphae appeared again at 21 dpi (Figure 5a, bottom panel). However, many chlamydospores were still observed in the *ΔFoMC69* mutant-inoculated roots at the same time (Figure 5b, bottom panel).

Chlamydospore numbers in the tomato roots at 7, 14, and 21 dpi were determined using a microscope. Chlamydospores of the wild-type and complementation strains increased from 7 dpi to 14 dpi, but decreased at 21 dpi. Incontrast, the number of chlamydospores in the *ΔFoMC69* mutant increased continuously during the inoculation test. Compared to the wild type and complementation strains, the number of chlamydospores in the *ΔFoMC69* mutant tended to be higher during infection, particularly at 7 dpi and 21 dpi (Figure 5c). 

To identify whether chlamydospores of *ΔFoMC69* were dead or alive, chlamydospores on the surface of the roots were stained with Evans blue at 21 dpi. No staining was observed, indicating that the chlamydospores on the root surface were alive, although they had not germinated (Supplementary Figure S4).

## 4. Discussion

In this study, we characterized *FoMC69* gene in Forl as a homolog of *MC69* required for pathogenicity in rice blast pathogen—*M. oryzae* [18]. The *ΔFoMC69* mutant of Forl did not affect root rot symptoms, unlike in the case of the wild-type strain, indicating the involvement of this gene in the full pathogenicity of Forl. The *ΔFoMC69* mutant of Forl did not reach the hypocotyl but was re-isolated from the root tissues. The number of colonies of the Δ*FoMC69* mutant was drastically lower than that of the wild-type strain, as determined by quantitative PCR. These results suggested the involvement of *FoMC69* of Forl in the invasion and colonization of tomato plant tissues. The *ΔMC69* mutant of the rice blast pathogen was reported not to affect radial growth, conidial formation, or appressorium formation. However, the mutant failed to develop invasive hyphae after appressorium formation [18]. The *MC69* homolog of *C. graminicola*, *CLU5a*, is also related to normal infection hyphae formation [19]. However, during a Forl infection, there are no specific infection sites or specific structures such as the appressorium [31].

In the current study, microscopic observation with WGA-FITC labeling revealed the infection process and importance of chlamydospores in Forl. Generally, chlamydospore formation is a survival strategy during undesirable conditions [3,4,32]. However, this study showed that chlamydospore formation was observed in plants inoculated with wild-type and *ΔFoMC69* mutants in the early stage. A similar result has been reported wherein chlamydospores were observed on the root surface at an earlier stage and within the vascular tissue at a later stage of infection in tomato [2]. Chlamydospores as inocula show higher virulence than conidia in Fol and *F. oxysporum* f. sp. *niveum* [33,34]. Ohara et al. demonstrated that *Ren1* mutant that formed abnormal conidia and normal chlamydospores still showed virulence in melon plants, suggesting no involvement of the microconidia and macroconidia in pathogenicity [35]. These observations demonstrate that chlamydospores may play important roles in the pathogenicity of the *F. oxysporum* species complex during infection. Moreover, chlamydospores of the *ΔFoMC69* mutant increased continuously during infection and were three times higher than those of the wild-type strain at 21 dpi. In addition, we observed that the chlamydospores were alive on the root surface, but did not germinate at 21 dpi. These results suggestthat the *FoMC69* gene is involved in the normal development and germination of chlamydospores.

This study showed that Fol also has *FoMC69* genes in its genome and few expressions of this gene was detected during infection. However, the *ΔFoMC69* mutant of Fol showed severe wilt symptoms similar to those of the wild-type strain, suggesting no involvement of the *FoMC69* gene in the pathogenicity of Fol. Fol causes wilt symptoms without root rot and forms a few chlamydospores in the root (data not shown), whereas Forl causes severe root rot symptoms with the formation of many chlamydospores. Moreover, Fol uses various effectors, such as *SIX* genes, to suppress PTI and colonize xylem vessels [36], but Forl has no *SIX* genes [14]. Forl produced many chlamydospores in the root at an early stage of infection, suggesting that it might avoid the plant’s innate immune response by converting mycelia to chlamydospores. The innate immune response may induce hypersensitive cell death, resulting in root rot. Activating an oxidative burst mediated by peroxidases and a cytochrome monooxygenase causes cell degeneration and necrosis in compatible interactions between Forl and tomato [37]. Further studies are required to clarify the relationship between chlamydospore formation and host oxidative burst in the early stages of Forl infection.

The function of *MC69* remains unclear. *MC69* of *M. oryzae* has also been characterized as *SPD1*, a suppressor of plant cell death. *SPD1* suppresses nep1- and Bax-induced plant cell death using agroinfiltration in *Nicotiana benthamina* [38]. However, Forl is a necrotrophic fungus that induces root rot in tomatoes, and *ΔFoMC69*-inoculated roots showed no symptoms. Thus, *FoMC69* may not be involved in suppressing plant cell death. Further research is required to understand the involvement of *MC69* in pathogenicity and whether a common function is conserved among the *MC69* homologs. 

*MC69* homologs are broadly conserved among members of the Pezizomycotina class, Sordariomycetes [19]. A conserved motif containing two cysteine residues presents high homology among all *MC69* homologs [18]. Therefore, MC69 may function as a core effector in Sordariomycetes. Consequently, we analyzed the role of *FoMC69* by quantifying the expression of defense-related genes. There were no differences between the *ΔFoMC69* mutant and wild-type strains (data not shown). Similarly, *MC69* of *M. oryzae* did not affect the expression of host defense-related genes or H_2_O_2_ accumulation, suggesting that the primary function of the protein is related to the structure or function of the fungus itself [18]. Eisermann et al. also hypothesized that *CLU5a*, an *MC69* homolog, may play a role in cell wall function, as *Δclu5a* mutants failed to develop penetration pegs and formed pressurized appressoria [19]. *FoMC69* was related to the normal development and germination of chlamydospores on the root surface. Our results strongly support the role of *MC69* homologs in the normality of cell structures during the infection process, although not with respect to functional effectors.

## Data Availability

Not applicable.

## Supplementary Materials

The following supporting information can be downloaded at https://www.mdpi.com/article/10.3390/pathogens11121433/s1, Figure S1: Southern blot analysis of *FoMC69* in Forl and Fol; Figure S2: Target gene disruption of *FoMC69*; Figure S3: Colony diameter and conidial formation of *ΔFoMC69* mutants, Figure S4: Evans blue staining of inoculated roots.
