# Supplementary material for: FoMC69 Gene in Fusarium oxysporum f. sp. radicis-lycopersici Is Essential for Pathogenicity by Involving Normal Function of Chlamydospores
Source: Pathogens. 2022 Nov 28;11(12):1433. doi: 10.3390/pathogens11121433 (PMC9784146; doi:10.3390/pathogens11121433)
Supplement: Supplementary file 1 [file pathogens-11-01433-s001.zip › pathogens-2049584-supplementary.pdf]

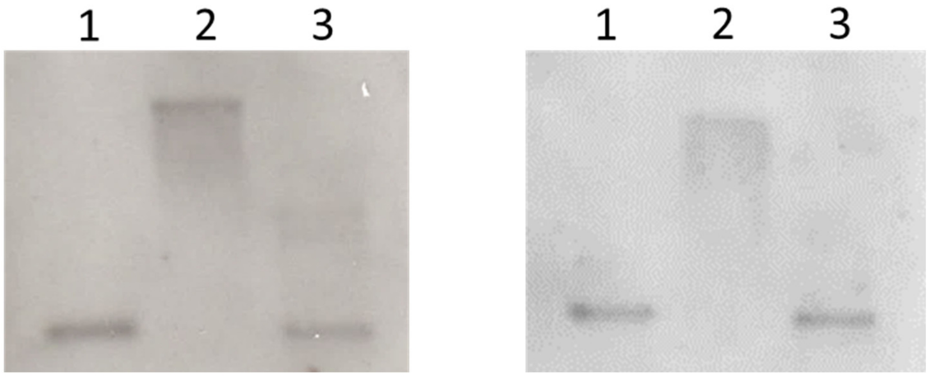

Figure S1. Southern blot analysis of *FoMC69* in Forl and Fol. Left panel indicates Forl, right panel is Fol. Fungal DNA were digested by restriction enzyme *Eco*RI (lane 1), *Sma*I (lane 2), and *Xho*I (lane 3) and were hybridized with *FoMC69* fragment as a probe.

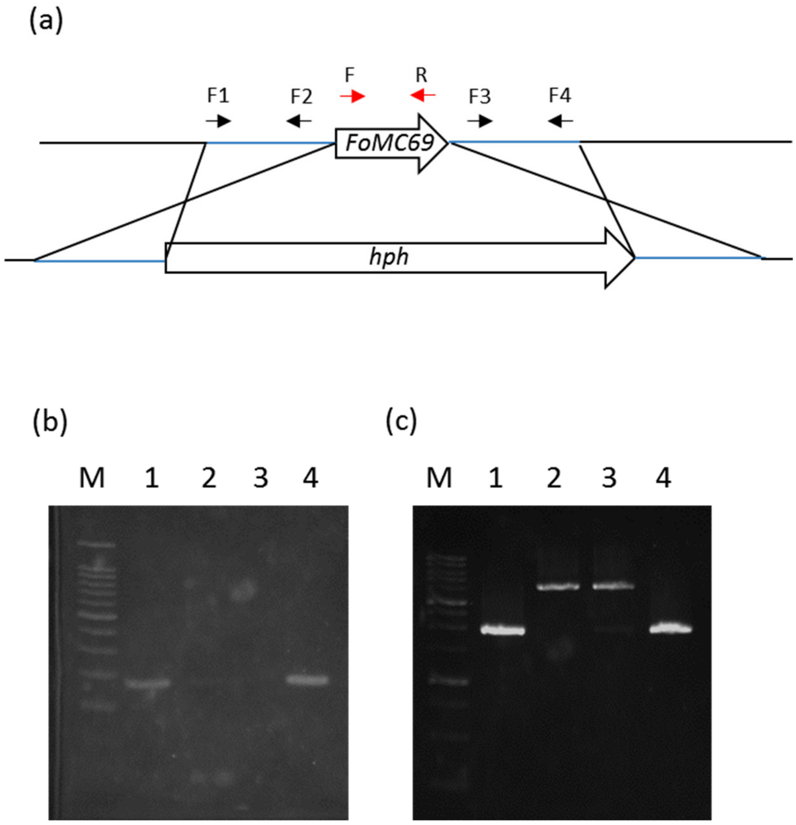

Figure S2. Target gene disruption of *FoMC69*. (a) Diagram of FoMC69 locus truncated by homologous recombination with hygromycin resistance cassette gene (*hph*). Arrows indicate primer position; F: FoMC69-Q-F, R: FoMC69-Q-R, F1: FoMC69-F1, F2: FoMC69-F2, F3: FoMC69-F3, F4: FoMC69-F4. (b) agarose gel electrophoresis of amplicon of FoMC69-Q-F/R primers. M: 100 bp ladder, 1: wild type, 2: *ΔFoMC69-1*, 3: *ΔFoMC69-2*, 4: *ΔFoMC69-1+FoMC69*. (c) Agarose gel electrophoresis of amplicon of FoMC69-F1/F4 primers. M: 100 bp ladder, 1: wild type, 2: *ΔFoMC69-1*, 3: *ΔFoMC69-2*, 4: *ΔFoMC69-1+FoMC69*.

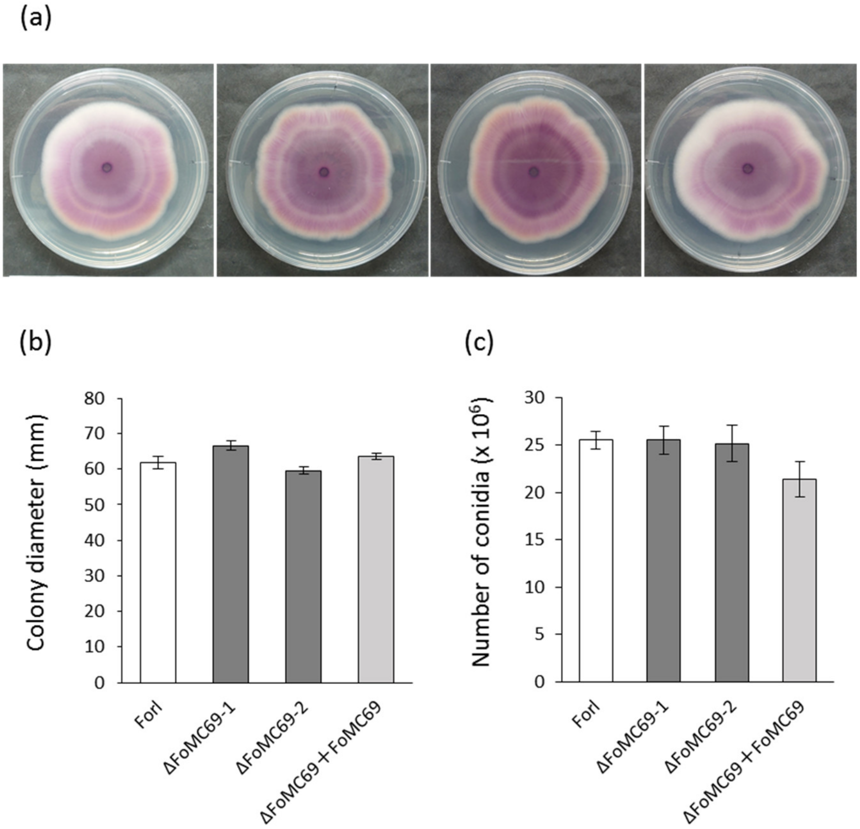

Figure S3. Colony diameter and conidial formation of *ΔFoMC69* mutants. (a) Colony morphology cultivated on potato dextrose agar (PDA) medium for 10 days. (b) Colony diameter cultivated on PDA medium for 10 days. (bc) Number of conidia formed on PDA medium for 14 days.

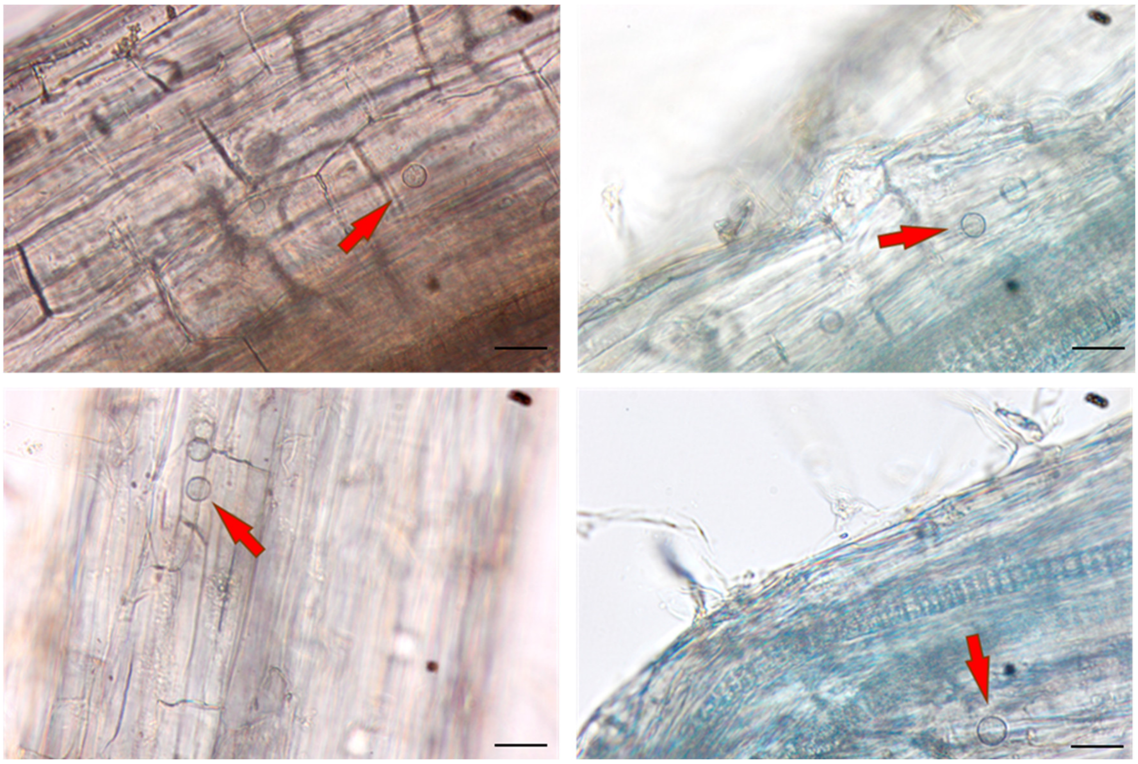

Figure S4. Evans blue staining of inoculated roots. Forl wild-type (top panels) and *ΔFoMC69* (bottom panels) at 7 day after inoculation (left panels) and 21 days after inoculation (right panels). Red arrows indicate chlamydospores. Bar are 20 μm.
